# Genotype–Phenotype Correlation in Neurofibromatosis Type 1: Evidence for a Mild Phenotype Associated with Splicing Variants Leading to In-Frame Skipping of *NF1* Exon 24 [19a]

**DOI:** 10.3390/cancers16132406

**Published:** 2024-06-29

**Authors:** Yunjia Chen, Yulong Fu, Magdalena Koczkowska, Tom Callens, Alicia Gomes, Jian Liu, William Bradley, Bryce Brown, Brandon Shaw, Daniela D’Agostino, Chuanhua Fu, Deeann Wallis

**Affiliations:** 1 Department of Genetics, University of Alabama at Birmingham, Birmingham, AL 35294, USA; magdalena.koczkowska@gumed.edu.pl (M.K.); tcallens@uabmc.edu (T.C.); agomes@uabmc.edu (A.G.); jianliu@uabmc.edu (J.L.); willb456@uab.edu (W.B.); ebfincher@uabmc.edu (B.B.); brandonshaw@uabmc.edu (B.S.); chuanhuafu@uabmc.edu (C.F.); dwallis@uab.edu (D.W.); 2 Department of Pathology, University of Alabama at Birmingham, Birmingham, AL 35294, USA; 3 3P-Medicine Laboratory, Medical University of Gdansk, 80-211 Gdansk, Poland; 4 Division of Medical Genetics, Departments of Medicine and Human Genetics, McGill University, Montreal, QC H3A 0G4, Canada; daniela.dagostino.med@ssss.gouv.qc.ca

**Keywords:** NF1, splicing variant, exon skipping, genotype–phenotype correlation

## Abstract

**Simple Summary:**

Although a large number of *NF1* variants have been cataloged in public databases, known NF1 genotype-phenotype correlations are still limited. Through a retrospective analysis of a large cohort of NF1 patients at the Medical Genomics Laboratory of the University of Alabama at Birmingham, we established a novel NF1 genotype-phenotype correlation. Specifically, NF1 patients with *NF1* exon 24 [19a] skipping typically exhibit a mild phenotype, characterized by the absence of severe NF1-specific clinical features, including neurofibromas. This newly established genotype-phenotype correlation will help clinicians improve the management of patients harboring *NF1* exon 24 [19a] skipping variants and provide a new therapeutic target for NF1 treatment.

**Abstract:**

Neurofibromatosis type 1 (NF1) is an autosomal dominant neurocutaneous disorder caused by loss-of-function variants in the *NF1* gene. As of 20 November 2023, over 5000 distinct pathogenic or likely pathogenic variants have been reported in public databases. However, only a few *NF1* genotype–phenotype correlations have been established so far. In this study, we present findings on 40 individuals with NF1, comprising 26 unrelated probands and 14 affected relatives, who carry one of nine *NF1* heterozygous pathogenic splicing variants, all of which result in the in-frame skipping of exon 24 [19a] (NM_000267.3:r.3114_3197del, p.Asn1039_Arg1066del). These variants include c.3114-2A>G, c.3114-1G>A, c.3196A>G, c.3197G>A, c.3197G>T, c.3197+1G>A, c.3197+1G>T, c.3197+2T>C, and c.3197+3A>T. Among individuals with these variants, none exhibit externally visible plexiform neurofibromas, histopathologically confirmed cutaneous or subcutaneous neurofibromas, symptomatic spinal neurofibromas, or symptomatic optic pathway gliomas. The most prevalent, and sometimes sole, clinical feature observed in this cohort is multiple café-au-lait macules, with or without skinfold freckles: 85% and 60.5% of the individuals display six or more café-au-lait macules and freckles, respectively. In comparison to established NF1 genotype–phenotype correlations, these patients demonstrate highly similar clinical presentations to those associated with the *NF1* pathogenic variant c.2970_2972del (p.Met992del), known for resulting in the mildest clinical features. Despite the generally mild phenotype, cognitive impairment, developmental delay, and/or learning difficulties are still observed in 33.3% of these patients, suggesting that learning challenges remain a prominent aspect of the phenotypic presentation in these individuals and necessitate specialized care. This newly established genotype–phenotype correlation will assist clinicians in improving the management of patients harboring *NF1* exon 24 [19a] skipping variants and provide a new therapeutic target for NF1 treatment.

## 1. Introduction

Neurofibromatosis type 1 (NF1; MIM: 162200) is an autosomal dominant neurocutaneous disorder with a prevalence of 1:2000–3000 live births [1,2,3]. The clinical manifestations observed in NF1-affected patients vary widely, with some being age-dependent. These features include multiple café-au-lait macules (CALMs), skinfold freckles, Lisch nodules, neurofibromas, optic pathway gliomas (OPGs), skeletal abnormalities, and learning difficulties [4].

NF1 is caused by loss-of-function variants in the tumor suppressor gene *NF1* (MIM: 613113), one of the genes with the highest spontaneous mutation rate [5]. As of 20 November 2023, more than 5000 distinct pathogenic or likely pathogenic variants have been reported in the ClinVar and Leiden Open Variation Database (LOVD). However, due to the variability in clinical presentation, the age dependency of most manifestations, and the wide distribution and allelic heterogeneity of *NF1* germline variants, only a few NF1 genotype–phenotype correlations have been established so far.

The constitutional *NF1* microdeletion, identified in approximately 4.7–13% of individuals with NF1, was initially recognized for its genotype–phenotype correlation with a severe form of the disease [6,7,8,9,10]. Patients with constitutional *NF1* microdeletions are characterized by dysmorphic facial features, intellectual disability and/or developmental delay, the early appearance of cutaneous neurofibromas, a higher tumor burden, and an increased lifetime risk for malignant peripheral nerve sheath tumors (MPNSTs) [11,12,13].

*NF1* missense variants at codons 844–848, 1276, and 1423 are also associated with severe phenotypes [14,15]. Specifically, pathogenic missense variants at codons 844–848 are associated with a high incidence of major superficial plexiform neurofibromas, symptomatic spinal neurofibromas, optic pathway gliomas, and/or skeletal abnormalities, as well as a pronounced predisposition to develop malignancies [14]. Pathogenic missense variants at p.Arg1276 and p.Lys1423 are associated with a high prevalence of symptomatic spinal neurofibromas and major superficial plexiform neurofibromas, respectively. Additionally, both variants are linked to cardiovascular abnormalities and Noonan-like phenotypes [15].

There are also *NF1* variants associated with mild phenotypes, including missense pathogenic variants at codons 1149 and 1809, as well as the 3-bp in-frame *NF1* deletion (c.2970_2972del, p.Met992del). These variants result in a clinical presentation lacking any externally visible plexiform, cutaneous, or subcutaneous neurofibromas. However, these variants, especially pathogenic missense variants at codons 1149 and 1809, are associated with a high incidence of Noonan-like features, including developmental delay and/or learning disabilities [15,16,17,18,19,20].

A substantial proportion (~30%) of pathogenic variants in the *NF1* gene affect mRNA splicing [21,22,23]. For splicing variants situated at or near the canonical splicing sites, one of the most common effects is the complete skipping of the whole exon, resulting from the disruption of the canonical splicing sites [24]. A review of *NF1* exon deletions and reported phenotypes, all LOVD 3.0 entries describing *NF1* exon deletions, and literature references has been previously compiled [25]. However, to our knowledge, no genotype–phenotype correlations related to *NF1* splicing variants or whole exon skipping have been established so far. In this study, we report nine different splicing variants from 40 patients, all of which lead to *NF1* exon 24 [19a] skipping, inducing very mild phenotypes. In addition to confirmed splicing effects through RNA-based testing, the pathogenicity of these variants was further validated by cell-based functional cDNA assays. This newly established genotype–phenotype correlation will be valuable for the clinical management and genetic counseling of patients harboring NF1 exon 24 [19a] skipping variants and provide a new therapeutic target for NF1 treatment.

## 2. Patients and Methods

### 2.1. Individuals and Phenotypic Data

A total of 40 individuals from 26 unrelated families, heterozygous for an *NF1* pathogenic variant that leads to exon 24 [19a] skipping, were included in this study. These patients were screened from approximately 14,000 NF1-affected individuals who were referred to the Medical Genomics Laboratory at the University of Alabama at Birmingham (UAB) for NF1 clinical genetic testing between 2003 and 2022 to establish or confirm the diagnosis of NF1 (hereafter referred to as the UAB cohort) and had positive genetic testing results. Any patients harboring variants that have been confirmed to result in *NF1* exon 24 [19a] skipping by the UAB Medical Genomics Laboratory were included in this study. Comprehensive *NF1* variant analysis was performed for 26 proband patients, while targeted gDNA-based Sanger sequencing analysis of *NF1* exon 24 [19a] was performed for 14 relatives. The phenotypic information for these individuals was mainly derived from the originally submitted phenotypic checklist form, as previously reported [18,25], and/or clinical notes submitted at the time when genetic testing was ordered. Updated phenotypic information was obtained for six of these patients. For individuals with missing information regarding a specific clinical feature, they were labeled as “not specified” (indicating that the information was either not checked on the originally submitted phenotypic checklist form or not specified in the clinical notes) or “unknown” (directly marked as unknown on the phenotypic checklist form). As a result, these individuals were excluded from the corresponding aggregated clinical data.

### 2.2. Comprehensive NF1 Molecular Analysis

Comprehensive *NF1* variant analysis includes gDNA-based Next-Generation Sequencing (NGS) and/or RNA-based Sanger sequencing, as well as copy number variant (CNV) analysis by multiplex ligation-dependent probe amplification (MLPA). The gDNA-based NGS analysis covers the entire coding region of the *NF1* gene, as well as intronic regions containing known deep intronic pathogenic variants [26]. The NGS test utilizes an extensively customized and optimized set of Agilent HaloPlex capture probes, followed by sequencing of overlapping amplicons within the regions of interest using 300 base pairs (bp) paired-end Illumina sequencing chemistry. Each coding exon plus at least 50 bp of flanking intronic sequence are simultaneously sequenced. RNA-based Sanger sequencing and CNV analysis by MLPA were performed as previously described [22,26,27]. Variants were described following the Human Genome Variation Society (HGVS) nomenclature standard and based on the *NF1* transcript isoform NM_000267.3 unless explicitly specified otherwise. The numbering of *NF1* exons was determined using both the NCBI numbering and legacy numbering, which was developed by the NF1 community. The legacy exon number was placed in square brackets after the NCBI exon number. The pathogenicity of variants was assessed by following recommendations from the American College of Medical Genetics and Genomics (ACMG) and the Association for Molecular Pathology (AMP) [28], as well as the ClinGen Sequence Variant Interpretation (SVI) Splicing Subgroup [29].

### 2.3. Splicing Assessment for Twelve NF1 Variants at Seven Positions (c.3114-2, c.3114-1, c.3196, c.3197, c.3197+1, c.3197+2, c.3197+3), Which Might Be Associated with Exon 24 [19a] Skipping but Not Detected in the UAB Cohort

In addition to the nine single nucleotide exchange variants (SNVs) leading to *NF1* exon 24 [19a] skipping identified in the UAB cohort, there are 12 other SNVs at c.3114-2, c.3114-1, c.3196, c.3197, c.3197+1, c.3197+2, c.3197+3, which also have the potential to induce *NF1* exon 24 [19a] skipping. A comprehensive splicing effect evaluation and pathogenicity classification following recommendations from ACMG/AMP and the ClinGen SVI Splicing Subgroup were performed on these variants. In silico splicing prediction tool SpliceAI was utilized to check the splicing effects of these variants. SpliceAI (https://spliceailookup.broadinstitute.org/ (as of 9 January 2024)) is a splice site prediction software based on a 32-layer deep neural network and has been recommended as the major splicing prediction tool by multiple ClinGen Variant Curation Expert Panel (VCEP) groups [30,31,32]. The settings for SpliceAI were configured as follows: hg19 genome version, max distance of 500, without selecting the check boxes for “masked scores” and “REF & ALT score column”. Additionally, publicly available databases, including the Leiden Open Variation Database (LOVD; as of 15 April 2024), the Human Gene Mutation Database (HGMD; as of 10 May 2023), and ClinVar (as of 15 April 2024), as well as data from 1000 Genomes and the Genome Aggregation Database (gnomAD; v4.0.0), were employed in the evaluation. Variants pathogenicity was determined based on recommendations from ACMG/AMP and the ClinGen SVI Splicing Subgroup [28,29].

### 2.4. Effects of NF1 Exon 24 [19a] Skipping on 3D Structure of Neurofibromin

3D structure homology modeling of human neurofibromin with the deletion from p.Asn1039 to p.Arg1066, due to *NF1* exon 24 [19a] skipping, was performed using SWISS-MODEL software (https://swissmodel.expasy.org/, as of 28 January 2024) based on the published neurofibromin dimer structure (closed state; PDB accession ID: 7PGR) [33]. An analysis was then performed utilizing UCSF Chimera (version 1.17.3) to compare the structure of the neurofibromin dimer in both closed and open states (PDB accession ID: 7PGR and 7PGT, respectively). Additionally, a comparative assessment was conducted between the newly modeled neurofibromin with the deletion and the neurofibromin dimer in the closed state.

### 2.5. Assessment of Expression/Stability and Activity of Mouse Neurofibromin Lacking 28 Amino Acids Encoded by Exon 24 in Cells

We utilized full-length cDNA plasmids representing several protein isoforms including wild-type (WT) mouse *Nf1* cDNA (NM_010897.2), *Nf1* cDNA lacking exon 24 (*NF1*:c.3114_3197), *Nf1* cDNA with the c.3827G>A, p.Arg1276Gln pathogenic variant, and a plasmid without cDNA (empty vector (EV)). These were individually transfected into *NF1*^−/−^ or null HEK293 cells. Subsequently, RAS-G-LISA assay and Western blot analysis for both WT and mutant neurofibromin isoforms were conducted. Detailed procedures for these assays, as well as cell culture, plasmid construction, and transfection, were previously described [25,34].

### 2.6. Statistical Analysis

A two-tailed Fisher’s exact test with *p* < 0.05 considered statistically significant was applied. The resulting *p* values were adjusted for multiple comparisons using the Benjamini–Hochberg (B-H) procedure with false discovery rates (FDR) at 0.05 and 0.01 [35]. These statistical analyses were performed with GraphPad software (https://www.graphpad.com/quickcalcs/contingency1/, as of 29 November 2023).

## 3. Results

### 3.1. Description of the Variants Leading to NF1 Exon 24 [19a] Skipping

**Figure 1:**
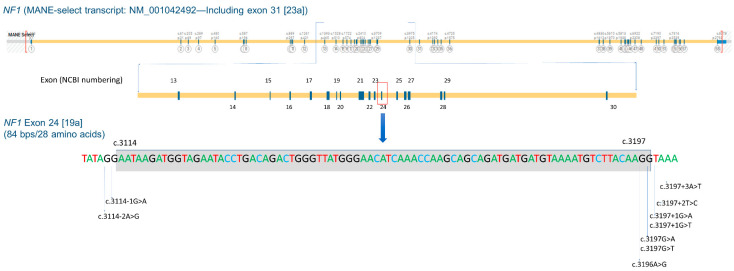
Schematic representation of the *NF1* gene (the MANE-select transcript NM_001042492), *NF1* exon 24 [19a] (c.3114_c.3197), and nine splicing variants that result in exon 24 [19a] skipping (c.3114-2A>G, c.3114-1G>A, c.3196A>G, c.3197G>A, c.3197G>T, c.3197+1G>A, c.3197+1G>T, c.3197+2T>C, and c.3197+3A>T).

**Figure 2:**
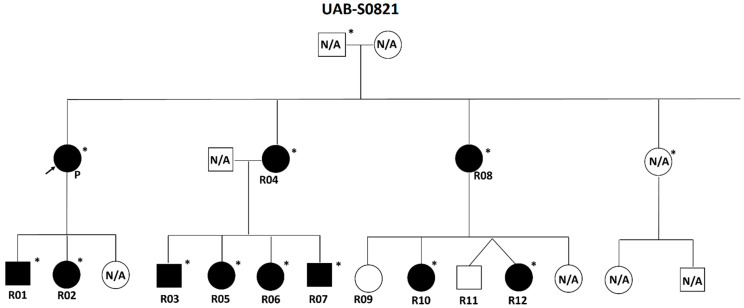
Pedigree of patients carrying the *NF1*:c.3197+3A>T pathogenic variant spanning two generations of a single family. Squares and circles indicate males and females, respectively. Filled symbols represent individuals with the *NF1*:c.3197+3A>T variant, open symbols labeled with “N/A” indicate individuals with uncertain status for the *NF1*:c.3197+3A>T variant, and open symbols without the label “N/A” indicate individuals without the *NF1*:c.3197+3A>T variant. The star marker (*) indicates that the patient presents with multiple CALMs with or without other NF1 clinical features. Specifically, the designation “P”, as well as the black arrow, refers to the proband (patient UAB-S0821-P, Table S1). The labels R01 to R08, R10, and R12 correspond to patients UAB-S0821-R01 to UAB-S0821-R08, UAB-S0821-R10, and UAB-S0821-R12, respectively (Table S1). R09, who presented with a single small CALM, and R11, displaying no NF1 features, underwent targeted testing for the *NF1*:c.3197+3A>T variant, yielding negative results.

*NF1* exon 24 [19a] is situated in the middle of the *NF1* gene and spans 84 bp (c.3114 to c.3197). The skipping of this exon results in the deletion of 28 amino acids from p.Asn1039 to p.Arg1066 (Figure 1). Nine different *NF1* heterozygous pathogenic splicing variants resulting in exon 24 [19a] skipping were identified in 40 individuals from 26 different families (Figure 1 and Table 1). These variants include two canonical splicing variants at the acceptor sites of exon 24 [19a] (c.3114-2A>G and c.3114-1G>A), three exonic splicing variants near the donor site of this exon (c.3196A>G, c.3197G>A, and c.3197G>T), three canonical splicing variants at the donor sites (c.3197+1G>A, c.3197+1G>T, and c.3197+2T>C), and one intronic splicing variant at the +3 position of this exon, namely c.3197+3A>T (Figure 1 and Table 1). The UAB Medical Genomics Laboratory has verified the splicing effect of eight out of nine variants using RNA-based (cDNA) Sanger sequencing (Supplementary Figure S1). The remaining variant, c.3197+1G>T, was confirmed by an external laboratory with the same methodology [36]. All nine variants have been reported in the ClinVar database, with several also being reported in LOVD and/or HGMD. Only two out of nine variants (c.3114-2A>G and c.3196A>G) were observed in gnomAD v4.0.0 with an extremely low frequency (1/1,435,698 and 1/1,455,704, respectively). In addition, family studies were conducted for seven of the 26 probands. Among them, five were confirmed to segregate NF1 features within their families. Notably, one variant (c.3197+3A>T) was found to segregate in 11 patients across two generations of a single family (Figure 2). Based on recommendations from ACMG/AMP and the ClinGen SVI Splicing Subgroup, all nine of these variants were classified as pathogenic (Table 1).

It is noteworthy that no other pathogenic or likely pathogenic variants or variants of uncertain clinical significance in the *NF1* gene were identified in the 26 probands. Additionally, comprehensive *SPRED1* gDNA-based analysis was performed for 15 out of the 26 proband patients, and no reportable variants were found (Table S1).

### 3.2. Clinical Characterization of the Patient Cohort

Detailed clinical descriptions of 40 individuals from 26 unrelated families who are heterozygous for one of nine different pathogenic *NF1* splicing variants that result in exon 24 [19a] skipping are presented in Table S1. All individuals in this study presented with a mild phenotype, and the major clinical features are limited to multiple CALMs, with or without skinfold freckles. Among these patients, 85% (34/40) have ≥6 CALMs, and 60.5% (23/38) are presenting with freckles. Specifically, among the individuals ≥ 9 years, 82.6% (19/23) and 72.7% (16/22) have ≥6 CALMs and freckles, respectively. No Lisch nodules (0/23), externally visible plexiform (0/31, with 0/18 for individuals ≥ 9 years), symptomatic spinal neurofibromas (0/33), or symptomatic OPG (0/37) were seen in this cohort. Furthermore, none of the patients presented with histopathologically confirmed cutaneous or subcutaneous neurofibromas (0/35 or 0/33, respectively, with 0/8 for individuals ≥ 19 years), except for one individual (UAB-S0821-R08), for whom a single suspected cutaneous neurofibroma was reported. The prevalence of skeletal abnormalities was 11.1% (4/36), with mild scoliosis observed twice and pectus carinatum and excavatum identified once in two individuals. Among the eight Noonan-like features considered in this study—namely, short stature, low-set ears, hypertelorism, midface hypoplasia, webbed neck, ptosis, down-slanting palpebral fissures, pectus abnormality, and pulmonic stenosis—only 1 out of 35 patients was reported to have two or more features (Patient UAB-S9876, with pectus excavatum, low-set ears, and midface hypoplasia), and none had pulmonic stenosis. Notably, one-third of patients in this study (33.3%, 13/39) exhibited cognitive impairment, developmental delay, and/or learning disabilities. Additionally, no patients presented with cardiovascular abnormalities. In terms of other tumor types, three patients were identified: one with a ganglioneuroblastoma (Patient UAB-N45811205), one with sebaceous adenomas on the nose (Patient UAB-S3276), and the last with a suspected retroperitoneal paraganglioma (UAB-S0821-R01). Lastly, following the revised NF1 diagnosis criteria [4], including the genetic testing results, 90% (36/40) of patients could be diagnosed with NF1.

### 3.3. Comparison of Clinical Features of the Studied Cohort with the Cohort of Individuals Heterozygous for NF1 p.Met992del, Cohorts of Individuals Carrying NF1 Missense Pathogenic Variants Affecting Codons 1809 and 844–848, and “Classic” NF1 Population

Detailed comparisons of clinical features between patients in the studied cohort and cohorts of individuals heterozygous for *NF1* p.Met992del, those carrying *NF1* missense pathogenic variants affecting codons 1809 and 844–848, as well as the “classic” NF1 population, are outlined in Table 2.

Remarkably, individuals in this study presented with a very mild phenotype, with no confirmed neurofibromas, optic pathway gliomas, or Lisch nodules. As a result, most clinical features listed in Table 2 did not exhibit statistically significant differences compared to cohorts of individuals with *NF1* p.Met992del and those with pathogenic missense variants affecting codons 1809, both of which were reported to present with a mild phenotype. This study also revealed a lower prevalence of Noonan-like features in comparison to individuals with pathogenic missense variants affecting codons 1809 (2.9% versus 31.1%, *p* = 0.0002, statistically significant after B-H correction at an FDR of 0.01; see Table 2 and Table S3). However, no statistical significance was observed for Noonan-like features when compared to the cohort of individuals with heterozygous *NF1* p.Met992del, likely due to the relatively small sample size of this study.

On the other hand, significant differences were found in several clinical features when compared to individuals harboring *NF1* missense pathogenic variants affecting codons 844–848 and the “classic” NF1 population. Specifically, individuals in this study showed a lower prevalence of Lisch nodules, externally visible plexiform neurofibromas, cutaneous neurofibromas, and skeletal abnormalities, including scoliosis, compared to those with missense pathogenic variants affecting codons 844–848 (*p* < 0.01, statistically significant after B-H correction at FDR 0.05; see Table 2 and Table S3). Furthermore, in contrast to the “classic” NF1 population, the occurrence of freckles, Lisch nodules, cutaneous neurofibromas, and subcutaneous neurofibromas was significantly less in the cohort of this study (*p* < 0.01, statistically significant after B-H correction at FDR 0.01; see Table 2 and Table S3).

### 3.4. Assessment of NF1 Variants at c.3114-2, c.3114-1, c.3196, c.3197, c.3197+1, c.3197+2, c.3197+3, Which Potentially Lead to Exon 24 [19a] Skipping but Have Not Been Reported

In the UAB cohort, a total of nine distinct variants located at seven positions have been identified to cause *NF1* exon 24 [19a] skipping (Figure 1 and Figure S1). These seven positions also exhibit an additional 12 potential single nucleotide substitutions that could lead to the same outcome but have not been observed in the UAB cohort (Table S2). Among these 12 substitutions, six have been documented in the literature [44] or databases (ClinVar, HGMD, and/or LOVD).

Using SpliceAI, we analyzed the potential splicing effects of these 12 substitutions. The results showed that nine of them have SpliceAI Donor/Acceptor Loss scores that are generally the same (between 0.85 and 1) as the nine variants identified in the UAB cohort (between 0.81 and 1). Based on the ACMG guidelines and recommendations from the ClinGen SVI Splicing Subgroup [28,29], these nine variants were classified as pathogenic or likely pathogenic. However, three of them have significantly lower SpliceAI Donor/Acceptor Loss scores (between 0.05 and 0.29) compared to the nine variants identified in the UAB cohort (Table S2). This suggests that these three substitutions might not lead to abnormal splicing. These three variants are c.3196A>C (SpliceAI Donor Loss score = 0.05), c.3196A>T (SpliceAI Donor Loss score = 0.29), and c.3197+3A>G (SpliceAI Donor Loss score = 0.08) (Table S2). Although SpliceAI prediction does not support c.3196A>T as a splicing variant, it was still classified as likely pathogenic due to the prediction of creating a premature stop codon, p.(Arg1066Ter) in the *NF1* mRNA. As no predicted splicing effect was observed, the potential synonymous variant c.3196A>C and the intronic variant c.3197+3A>G were considered variants of uncertain clinical significance, following the ACMG guidelines and recommendations from the ClinGen SVI Splicing Subgroup.

### 3.5. 3D Structure Change of Neurofibromin Due to NF1 Exon 24 [19a] Skipping

While in the past, individual domains of neurofibromin have been crystalized, only recently have several groups utilized cryo-EM to resolve the structure of full-length neurofibromin [33,45,46,47]. Figure S2 depicts the region of p.Asn1039_Arg1066 in the 3D structures of the human neurofibromin dimer, highlighting its relative position to the key functional domains, i.e., the GAP-related domain (GRD) and Sec14-PH domain. The region spanning p.Asn1039_Arg1066 is situated at the bottom of the entire neurofibromin dimer, while the GRD domain and Sec14-PH domain, crucial for interacting with RAS either directly or indirectly, are situated at the top of the neurofibromin dimer. Additionally, analysis of the structures in both “open” and “closed” states reveals that p.Asn1039_Arg1066 neither directly engages in the interaction between the GRD domain and RAS nor undergoes structural changes induced by this interaction. Figure S3 illustrates the homologous modeling structure of neurofibromin with the deletion of p.Asn1039_Arg1066 due to *NF1* exon 24 [19a] skipping. A comparison with the full-length structure demonstrates that the deletion of p.Asn1039_Arg1066 primarily affects itself and the adjacent residues, including p.Phe1037_Arg1038 and p.Asp1067_Met1073, without noticeably impacting the surrounding regions and the general structure of the neurofibromin.

### 3.6. Mutant Neurofibromin Levels and Ras Activity in HEK293 Cells

To determine the functional effects of skipping exon 24 [19a] on *NF1* protein (neurofibromin), we created and tested *Nf1* cDNAs coding for *NF1* isoforms with deletion of exon 24 [19a]. Our group and others have used such models to explore how *NF1* variants impact Ras signaling [25,34,48,49,50,51]. We used synthetic gene fragments to create this deletion and cloned it into a mouse *Nf1* cDNA plasmid. All clones were validated by sequencing the entire cDNA region, and all isoforms representing the variants were evaluated in at least three different functional assays of the NF1-Ras-mitogen-activated protein kinase (MAPK) signaling pathway.

First, we determined the level of neurofibromin in *NF1* null (−/−) HEK293 cells when transiently transfected with a constant amount of cDNA (1 μg). A representative Western blot probed with neurofibromin antibody is shown in Figure S5A, as are actin (used as loading control), pERK, and ERK blots. A minimum of three separate experiments were quantified and are depicted in Figure S5B as NF1/actin; all data are normalized to the WT cDNA such that data can be combined across experiments and blots. Loss of exon 24 led to significant decreases in neurofibromin levels in comparison with WT control via t-test (*p* < 0.05 and indicated with an asterisk in Figure S5B). Known pathogenic variant c.3827G>A, p.Arg1276Gln is also used as a comparator as previous data have indicated that it expresses near WT levels [49].

Second, we evaluated the ability of these shortened Nf1 protein constructs to regulate levels of GTP-Ras (Figure S5C). GTP-Ras levels of all mutant protein isoforms were statistically compared by t-test with that of empty vector (EV) plasmid with no cDNA insert. While WT cDNA is significantly more active than EV (*p* < 0.05) as it is able to repress Ras-GTP levels, the protein isoform lacking exon 24 was not more active than EV and did not display any ability to suppress levels of GTP-Ras. We have also included data from the known pathogenic variant p.Arg1276Gln, which is known to lose the ability to activate Ras’s GTPase in this assay [49].

Third, we evaluated downstream MAPK signaling focusing on pERK/ERK ratios (Figure S5A,D) as a second indication of the function of NF1 GRD-mediated GAP function. All samples were normalized to the WT protein isoform, and pERK/ERK levels of all mutant isoforms were compared with that of EV by t-test. Isoforms lacking exon 24 or containing the p.Arg1276Gln variant were unable to suppress levels of pERK through GRD-mediated GTPase activity on upstream Ras.

## 4. Discussion

*NF1* is one of the most mutable genes in the human genome, with more than 5000 different pathogenic or likely pathogenic variants documented in public databases. Despite this wealth of data, only a few genotype–phenotype correlations have been established due to variability in clinical presentation, age dependency of most manifestations, the timing and number of second hits in specific cells, and the wide *NF1* allelic heterogeneity. Establishing new genotype–phenotype correlations would be valuable for the clinical management and genetic counseling of NF1-affected patients and may provide new therapeutic targets for NF1 treatment [25,52].

In this study, we present 40 individuals who are heterozygous for one of the nine variants leading to the skipping of *NF1* exon 24 [19a]. These nine variants have been reported in the ClinVar, LOVD, and/or HGMD databases, with five being classified as pathogenic or likely pathogenic. The splicing effects of all nine variants have been confirmed by RNA-based testing. None of the nine variants are present in the normal population database, such as gnomAD, or have a very low frequency (<0.000001) if present. Furthermore, an intronic variant c.3197+3A>T has been confirmed to segregate with NF1 features in a family of 11 patients from two generations, including the proband’s two sisters, as well as their eight children (Figure 2). All 11 patients exhibit multiple CALMs with or without other NF1 features. Additionally, one niece and one nephew of the proband (Figure 2, UAB-S0821-R09 and UAB-S0821-R11), who do not manifest any NF1 clinical features, were tested negative for the familial variant. Considering all available evidence and based on the ACMG guidelines and recommendations from the ClinGen SVI splicing variant group [27,28], all these nine variants have been classified as pathogenic (Table 1).

Neurofibromas are a hallmark of NF1, with over 50% of NF1 patients (over 90% for adult patients) presenting with cutaneous and subcutaneous neurofibromas [14,43,53,54], 15–30% presenting with externally visible plexiform neurofibromas [40,43,53,55,56], and approximately 2% presenting with symptomatic spinal neurofibromas [53,57]. Interestingly, in this study, none of the 40 individuals developed histopathologically confirmed cutaneous/subcutaneous neurofibromas (0/8 aged ≥19 years), externally visible plexiform neurofibromas (0/13 aged ≥9 years), or symptomatic spinal neurofibromas (0/33). Only one patient (UAB-S0821-R08, aged >26) was noted to have a potential single cutaneous neurofibroma pending histopathological confirmation. The lack of neurofibromas in this cohort is similar to our previously reported cohorts of p.Met992del, p.Met1149, and p.Arg1809 missense variants, all of which have a milder phenotype compared to the classic NF1 population [15,16,18,19].

Noonan-like features represent noteworthy clinical manifestations in certain NF1 patients, particularly those harboring missense variants at p.Arg1809. In this study, a significantly lower percentage of patients exhibited Noonan-like features compared to the *NF1* p.Arg1809 missense variants cohort (2.9% versus 31.1%, *p* = 0.0002, statistically significant after B-H correction at an FDR of 0.01; see Table 2 and Table S3). It is noteworthy that no statistically significant differences were observed in any paired comparisons between this studied cohort and the *NF1* p.Met992del cohort. Therefore, the studied cohort closely resembles the *NF1* p.Met992del cohort, which is associated with the mildest phenotype in known cohorts with evident genotype–phenotype correlations.

Patients in this study only exhibit CALMs with or without freckles. However, these dermatological manifestations are not exclusive to NF1; for instance, Legius syndrome (MIM: 611431) also prominently features CALMs, although with distinct molecular pathogenesis involving pathogenic variants in the *SPRED1* gene (MIM: 609291). Consequently, distinguishing between these conditions solely based on clinical observations is challenging, particularly in the absence of family studies. In 2021, clinical testing results were integrated into the revised NF1 diagnostic criteria [4], effectively improving clinical NF1 diagnosis. Indeed, without considering clinical testing results, 35% (14/40) of patients in this study did not meet the revised NF1 diagnosis criteria, whereas when clinical testing results were taken into account, only 10% (4/40) of individuals did not meet the criteria. This underscores the importance of genetic testing in the clinical diagnosis of NF1 patients, particularly those with a mild phenotype.

To investigate the impact of the mutant neurofibromin lacking the 28 amino acids from Asn1039 to Arg1066, resulting from exon 24 [19a] skipping, on the neurofibromin 3D structure, we performed homology modeling for the mutant neurofibromin. This was based on the resolved structure of the neurofibromin dimer obtained from cryo-EM (PDB ID: 7PGR) [33]. The neurofibromin deletion region (p.Asn1039_Arg1066) is located at the bottom region of the neurofibromin dimer, spatially distant from crucial known functional domains of neurofibromin, especially the GRD and SEC14-PH domains that reside at the top of the dimer (Figure S2A,B). Furthermore, when comparing the open (interacting with RAS) and closed (without interacting with RAS) states of the neurofibromin dimer, no noticeable change was observed in the structure of p.Asn1039_Arg1066, suggesting that this region does not directly participate in the integration of GRD and RAS (Figure S2E,F). Most importantly, based on the homologous modeling results, the deletion of Asn1039_Arg1066 has no noticeable impacts on the overall neurofibromin structure (Figure S3A,B). The deletion only removes part of two alpha-helix loops at the bottom of the dimer and leads to a structural change in a few amino acids flanking this region (p.Phe1037_Arg1038 and p.Asp1067_Met1073) (Figure S3C–E). Additionally, the deletion of the single amino acid p.Met992, which also leads to a similar mild phenotype, occurs at an adjacent alpha-helix loop (Figure S4), suggesting that the deletion of part of these loops does not significantly interfere with the overall function of neurofibromin.

To further evaluate the potential effects of losing 28 amino acids encoded by *NF1* exon 24 [19a], we examined the mutant neurofibromin levels and Ras activity in cells (Figure S5). Despite the mutant isoform lacking exon 24 retaining some neurofibromin expression, which is significantly less than WT levels, it is unable to repress GTP-Ras levels or pERK/ERK ratios. While these results confirmed the loss of Ras-signaling inhibition and likely the pathogenicity of these splicing variants, they could not provide a clear explanation for why the mutant neurofibromin results in a mild phenotype. Similar results have been observed in another missense variant at p.Met1149 (p.Met1149Val), also leading to a mild phenotype [34]. These findings suggest that the current cell-based function studies alone may not elucidate all genotype–phenotype correlations and/or indicate that *NF1* functions outside of Ras modulation may contribute to the phenotype. Notably, Ras signaling assays are not recognized by the NF1 Variant Curation Expert Panel (VCEP) for variant interpretation, as a systematic study has not ever been conducted to set thresholds to define benign versus pathogenic variants.

Currently, treatment options for NF1 are limited. Gene-targeted therapies for NF1, aimed at increasing levels of functional neurofibromin in cells, are being investigated [58,59]. This study, highlighting a mild phenotype caused by in-frame skipping of *NF1* exon 24 [19a], offers a novel therapeutic target for NF1 patients with severe symptoms resulting from pathogenic variants in this exon. This study suggests that loss of exon 24 [19a] results in a shortened protein with some residual NF1 function that is enough to prevent the tumorigenic and/or “severe” phenotypes associated with NF1. As such, patients with a more deleterious pathogenic variant within exon 24 [19a] could potentially be treated with an exon skipping approach in which exon 24 [19a] is removed and *NF1* expression from that allele is restored. Based on our internal database at the Medical Genomics Laboratory, approximately 37 of 12,500 (0.3%) unrelated patients who have a positive *NF1* genetic testing result have pathogenic or likely pathogenic variants within exon 24 [19a] (Table S4). Exon skipping approaches have been successful for other rare pediatric diseases, including Duchenne’s muscular dystrophy. Exon skipping approaches for NF1 are currently under investigation, particularly for exons 17 and 52 [25].

This study also has limitations. Forty out of approximately 14,000 NF1-affected patients with positive genetic testing results in the UAB cohort were identified to harbor a single splicing variant, resulting in *NF1* exon 24 [19a] skipping. However, no single exon deletions encompassing *NF1* exon 24 [19a] were identified in our cohort of NF1 patients or public databases and have not been reported in the literature. Additionally, a majority of patients requested genetic testing before puberty and were lost to follow-up afterward; thus, there is a relatively small number of adult patients (eight individuals aged ≥19 years). The limited size of the dataset often results in wider confidence intervals for statistics. Therefore, for specific age-dependent NF1 features such as cutaneous neurofibromas, which typically manifest after puberty, it cannot be entirely ruled out that a small percentage of patients may develop them at a later age. Further studies on a larger dataset of adult NF1 patients are needed to confirm these findings.

## 5. Conclusions

Our study demonstrates that NF1 patients with *NF1* exon 24 [19a] skipping typically exhibit a mild phenotype characterized by the absence of severe NF1-specific clinical features, including neurofibromas. However, approximately 33.3% of these patients still present with cognitive impairment, developmental delay, and/or learning disabilities, which require specialized care. This newly established genotype–phenotype correlation will assist clinicians in improving the management of patients harboring *NF1* exon 24 [19a] skipping variants and provide a new therapeutic target for NF1 treatment.

## Figures and Tables

**Table 1:** Nine *NF1* splicing variants in the UAB cohort resulting in *NF1* exon 24 [19a] skipping (NM_000267.3:r.3114_3197del, p.Asn1039_Arg1066del).

Variant (cDNA Level)	Number of Patients in the UAB Cohort	Confirmed by RNA-Based Testing (Number of RNA Tests)	1000G	gnomAD	LOVD	ClinVar	HGMD	Evidence	Classification of Pathogenicity
c.3114-2A>G	7	Yes (5; this study and PMID: 24789688)	0	1 (in 1435698)	2 (Pathogenic/NA)	Present (Likely pathogenic)	Present (DM)	PVS1 (RNA) ^a^ + PS4_strong ^b^	Pathogenic
c.3114-1G>A	4	Yes (3; this study)	0	0	0	Present (Likely pathogenic)	0	PVS1 (RNA) + PS4_strong + PM2 ^e^	Pathogenic
c.3196A>G	5	Yes (3; this study)	0	1 (in 1455704)	0	Present (VUS)	0	PVS1 (RNA) + PS4_strong	Pathogenic
c.3197G>A	2	Yes (1; this study)	0	0	4 (Pathogenic; including 1 de novo)	Present (Conflicting: Likely pathogenic/VUS)	Present (DM)	PVS1 (RNA) + PS4_moderate ^c^ + PM6 ^f^	Pathogenic
c.3197G>T	2	Yes (1; this study)	0	0	0	Present (VUS)	0	PVS1 (RNA) + PS4_supporting ^d^ + PM2	Pathogenic
c.3197+1G>A	3	Yes (5; this study and PMID: 10607834, 18546366)	0	0	1 (Pathogenic; de novo)	Present (Pathogenic/Likely pathogenic)	Present (DM)	PVS1 (RNA) + PS4_strong + PM2 + PM6	Pathogenic
c.3197+1G>T	1	Yes (1; PMID: 18546366)	0	0	3 (Pathogenic; including 1 de novo)	Present (Pathogenic)	Present (DM)	PVS1 (RNA) + PM2 + PM6	Pathogenic
c.3197+2T>C	2	Yes (1; this study)	0	0	2 (Pathogenic)	Present (Pathogenic)	0	PVS1 (RNA) + PS4_suppoorting + PM2	Pathogenic
c.3197+3A>T	14	Yes (2; this study)	0	0	0	Present (VUS)	0	PVS1 (RNA) + PS4_strong + PP1_strong ^g^ + PM2	Pathogenic

1000G, 1000 Genomes; gnomAD, the Genome Aggregation Database; HGMD, the Human Gene Mutation Database; LOVD, the Leiden Open Variation Database; VUS, variant of uncertain clinical significance; UAB, the University of Alabama at Birmingham. ^a^ PVS1 (RNA): the splicing effect has been confirmed in at least one NF1-affected patient, based on recommendations from ClinGen SVI subgroup. ^b^ PS4_strong: the variant has been identified in 8~15 unrelated NF1 patients (or the splicing effect has been confirmed in ≥2 NF1 patients). ^c^ PS4_moderate: the variant has been identified in 4~7 unrelated NF1 patients. ^d^ PS4_supporting: the variant has been identified in 2~3 unrelated NF1 patients. ^e^ PM2: absence from the gnomAD database v4.0.0. ^f^ PM6: assumed de novo without confirmation of paternity and maternity. ^g^ PP1_strong: co-segregation with NF1 in ≥2 families, including ≥7 affected family members. According to the ACMG/AMP guidelines, the inclusion of the code PVS1 (RNA), along with 1, strong level evidence, or 2, moderate/supporting level evidence, results in the pathogenicity classification of “pathogenic”.

**Table 2:** Comparison of clinical features of the studied cohort with the cohort of individuals heterozygous for *NF1* p.Met992del, cohorts of individuals carrying *NF1* missense pathogenic variants affecting codons 1809 and 844–848, and “classic” NF1 population.

NF1 Feature	N (%)					*p* Value (2-Tailed Fisher’s Exact Test)			
	Skipping Exon 24 [19a]	p.Met992del ^a^	p.Arg1809 ^b^	aa 844–848 ^c^	Previously Reported NF1 Cohorts ^d^	Skipping Exon 24 [19a] vs. p.Met992del	Skipping Exon 24 [19a] vs. p.Arg1809	Skipping Exon 24 [19a] vs. aa 844–848	Skipping Exon 24 [19a] vs. “Classic” NF1
≥6 CALMs	34/40 (85.0)	165/182 (90.7)	157/169 (92.9)	130/157 (82.8)	1537/1728 (89)	0.2662	0.1211	0.8174	0.4415
Skinfold freckling	23/38 (60.5)	105/171 (61.4)	95/161 (59)	104/144 (72.2)	1403/1667 (84.2)	1	1	0.1699	0.0005 ** ↘
Lisch nodules	0/23 (0)	16/139 (11.5)	12/120 (10)	42/98 (42.9)	729/1237 (58.9)	0.1307	0.0768	<0.0001 ** ↘	<0.0001 ** ↘
Major external plexiform neurofibromas ^e^	0/18 (0)	0/125 (0)	0/105 (0)	36/92 (39.1)	120/648 (18.5)	1	1	0.0006 ** ↘	0.0554
Cutaneous neurofibromas ^f^	0/8 (0) ^g^	0–1/57 (0–1.8) ^h^	0/57 (0)	47/69 (68.1)	656/723 (90.7)	1	1	0.0003 ** ↘	<0.0001 ** ↘
Subcutaneous neurofibromas ^f^	0/8 (0)	0–3/36 (0–8.3) ^h^	0–5/57 (0–8.8) ^h^	33/65 (50.8)	297/515 (57.7)	1	1	0.0068 * ↘	0.0011 ** ↘
Symptomatic spinal neurofibromas	0/33 (0)	1/165 (0.6)	0/76 (0)	13/127 (10.2)	36/2058 (1.8)	1	1	0.0719	1
Symptomatic OPGs ^i^	0/37 (0)	0/170 (0)	0/139 (0)	12/136 (8.8)	64/1650 (3.9)	1	1	0.0721	0.3981
Skeletal abnormalities	4/36 (11.1)	30/172 (17.4)	21/126 (16.7)	48/144 (33.3)	144/948 (15.2)	0.4609	0.6014	0.0076 * ↘	0.6386
Scoliosis ^f^	2/36 (5.6)	7/57 (12.3)	6/48 (12.5)	20/64 (31.3)	51/236 (21.6)	0.4742	0.4566	0.0025 * ↘	0.0229 ↘
Cognitive impairment, developmental delay, and/or learning disabilities	13/39 (33.3)	58/176 (33)	80/159 (50.3)	56/138 (40.6)	190/424 (44.8)	1	0.0731	0.461	0.1811
Noonan-like features ^j^	1/35 (2.9)	19/166 (11.5)	46/148 (31.1)	10/134 (7.5)	57/1683 (3.4)	0.2099	0.0002 ** ↘	0.4629	1

Statistically significant *p* values with an FDR of 0.05 (indicated by *) and 0.01 (indicated by **) were observed after correction for multiple testing using the Benjamini–Hochberg (B-H) procedure (see details in Table S3). Following the B-H correction, *p* ≤ 0.0076 and *p* ≤ 0.0011 remained statistically significant at FDRs of 0.05 and 0.01, respectively. The black down arrows denote a decrease in prevalence in the study cohort compared to other cohorts. CALMs, café-au-lait macules; NF1, neurofibromatosis type 1; OPG, optic pathway glioma. ^a^ Based on data from Upadhyaya et al. (2007) [16] and Koczkowska et al. (2019) [19]. ^b^ Based on data from Pinna et al. (2015) [17], Rojnueangnit et al. (2015) [18], Ekvall et al. (2014) [37], Nyström et al. (2009) [38], and Santoro et al. (2015) [39]. ^c^ Based on data from Koczkowska et al. (2018) [14]. ^d^ Previous NF1 cohorts used for the comparison: Huson, Harper, and Compston (1988) [40]; Huson, Compston, Clark et al. (1989) [5]; Huson, Compston, and Harper (1989) [41]; Listernick, Charrow, Greenwald, and Mets (1994) [42]; and Friedman and Birch (1997) [43]. ^e^ In individuals ≥9 years old. ^f^ In individuals ≥19 years old. ^g^ One individual was noted to have a possible cutaneous neurofibroma without any confirmation. ^h^ Individuals with few (2–6) cutaneous or subcutaneous “neurofibromas”; none were biopsied, and therefore, none have been histologically confirmed. ^i^ The absence of symptomatic OPGs was determined by ophthalmological examination and/or by magnetic resonance imaging (MRI). ^j^ An individual was classified as having a Noonan-like phenotype when at least two of the following features were present: short stature, low-set ears, hypertelorism, midface hypoplasia, webbed neck, and/or pulmonic stenosis.

## Data Availability

All reported variants identified by our lab, as well as corresponding patients’ clinical features, have been included in this manuscript (see Table 1, Table 2 and Tables S1–S4 for details).

## Supplementary Materials

The following supporting information can be downloaded at https://www.mdpi.com/article/10.3390/cancers16132406/s1, Figure S1: Sanger sequencing results of eight *NF1* variants that result in exon 24 [19a] skipping; Figure S2: Location of the region of *NF1*:p.Asn1039_Arg1066 (deleted due to *NF1* exon 24 [19a] skipping), as well as flanking residues (p.Gln1033_Arg1038 and p.Asp1067_Met1073), in the 3D structures of human neurofibromin dimers, depicted by UCSF Chimera; Figure S3: The potential effect of the deletion of the region of p.Asn1039_Arg1066 on the 3D structures of human neurofibromin dimers; Figure S4: Location of Met992 (highlighted in red) and the region of *NF1*:p.Asn1039_Arg1066 (highlighted in purple), as well as flanking residues p.Gln1033_Arg1038 (highlighted in yellow) and p.Asp1067_Met1073 (highlighted in green), in the 3D structures of human neurofibromin dimers, depicted by UCSF Chimera; Figure S5: Functional analysis of mNf1 isoforms with selected variants: NF1 protein expression and RAS activity, Original western blots are presented in File S1; Table S1: Clinical details for 40 individuals from the UAB cohort with one of nine different splicing variants that lead to *NF1* exon 24 [19a] skipping; Table S2: Other *NF1* variants at c.3114-2, c.3114-1, c.3196, c.3197, c.3197+1, c.3197+2, c.3197+3, which might result in exon 24 [19a] skipping; Table S3: List of all adjusted *p* values from Table 2 after applying the Benjamini-Hochberg correction for multiple testing with false discovery rates (FDR) at 0.05 and 0.01; Table S4: Thirty-seven unrelated probands harboring pathogenic or likely pathogenic variants within *NF1* exon 24 [19a] from the UAB Medical Genomics Lab.
