# Supplementary material for: Genotype–Phenotype Correlation in Neurofibromatosis Type 1: Evidence for a Mild Phenotype Associated with Splicing Variants Leading to In-Frame Skipping of NF1 Exon 24 [19a]
Source: Cancers (Basel). 2024 Jun 29;16(13):2406. doi: 10.3390/cancers16132406 (PMC11240586; doi:10.3390/cancers16132406)
Supplement: Supplementary file 1 [file cancers-16-02406-s001.zip › File S1-Full Blots with marker lables.pptx]

## Slide 1
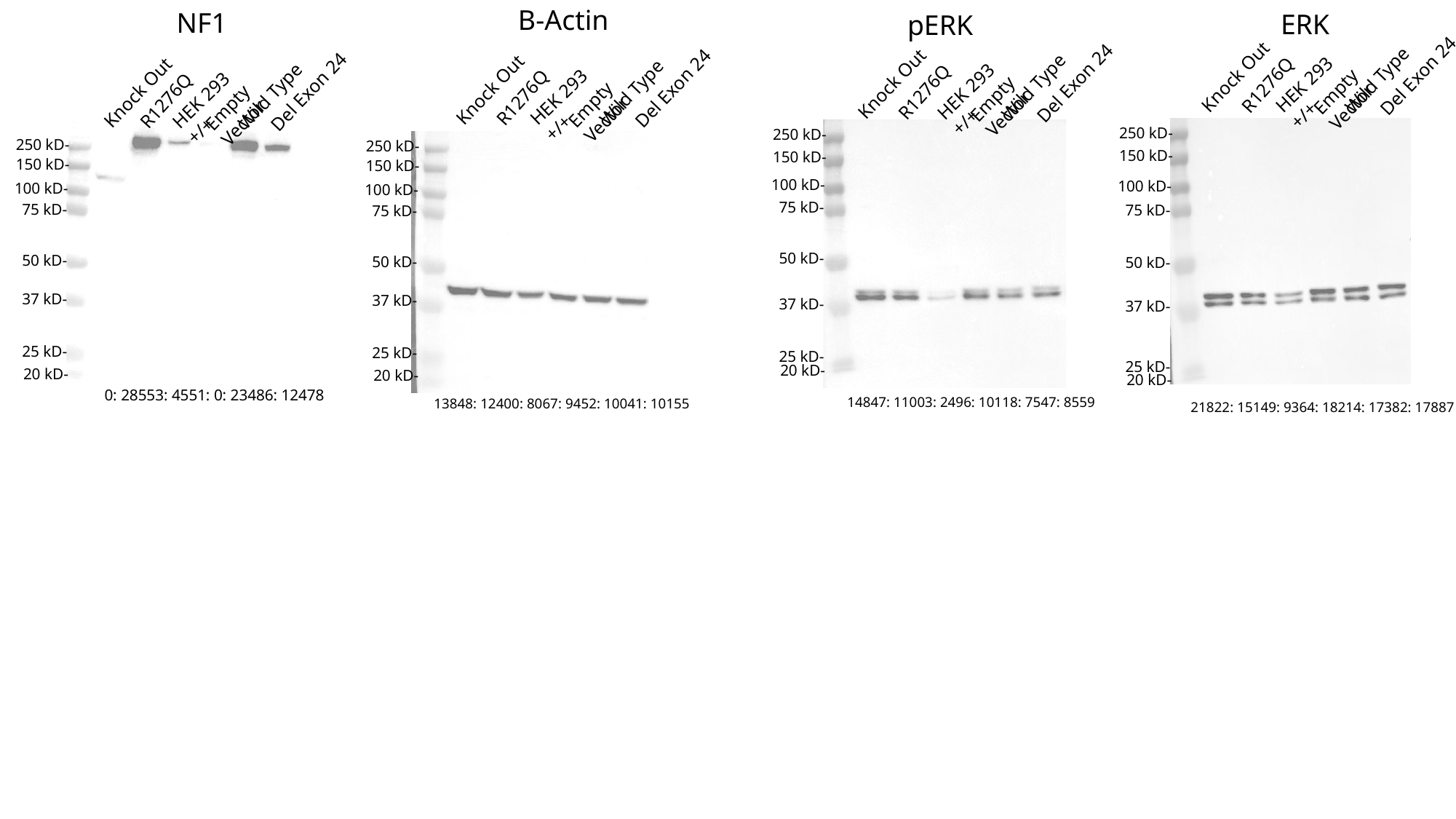

B-Actin
NF1
ERK
pERK
Wild Type
Empty Vector
Del Exon 24
R1276Q
Knock Out
HEK 293 +/+
250 kD-
150 kD-
100 kD-
75 kD-
50 kD-
37 kD-
25 kD-
20 kD-
Wild Type
Empty Vector
Del Exon 24
R1276Q
Knock Out
HEK 293 +/+
250 kD-
150 kD-
100 kD-
75 kD-
50 kD-
37 kD-
25 kD-
20 kD-
Wild Type
Empty Vector
Del Exon 24
R1276Q
Knock Out
HEK 293 +/+
Wild Type
Empty Vector
Del Exon 24
R1276Q
Knock Out
HEK 293 +/+
250 kD-
150 kD-
100 kD-
75 kD-
50 kD-
37 kD-
25 kD-
20 kD-
250 kD-
150 kD-
100 kD-
75 kD-
50 kD-
37 kD-
25 kD-
20 kD-
0: 28553: 4551: 0: 23486: 12478
14847: 11003: 2496: 10118: 7547: 8559
13848: 12400: 8067: 9452: 10041: 10155
21822: 15149: 9364: 18214: 17382: 17887
