# Supplementary material for: Genotype–Phenotype Correlation in Neurofibromatosis Type 1: Evidence for a Mild Phenotype Associated with Splicing Variants Leading to In-Frame Skipping of NF1 Exon 24 [19a]
Source: Cancers (Basel). 2024 Jun 29;16(13):2406. doi: 10.3390/cancers16132406 (PMC11240586; doi:10.3390/cancers16132406)
Supplement: Supplementary file 1 [file cancers-16-02406-s001.zip › Supp. Figures S1-S5.pdf]

Supplementary Figures

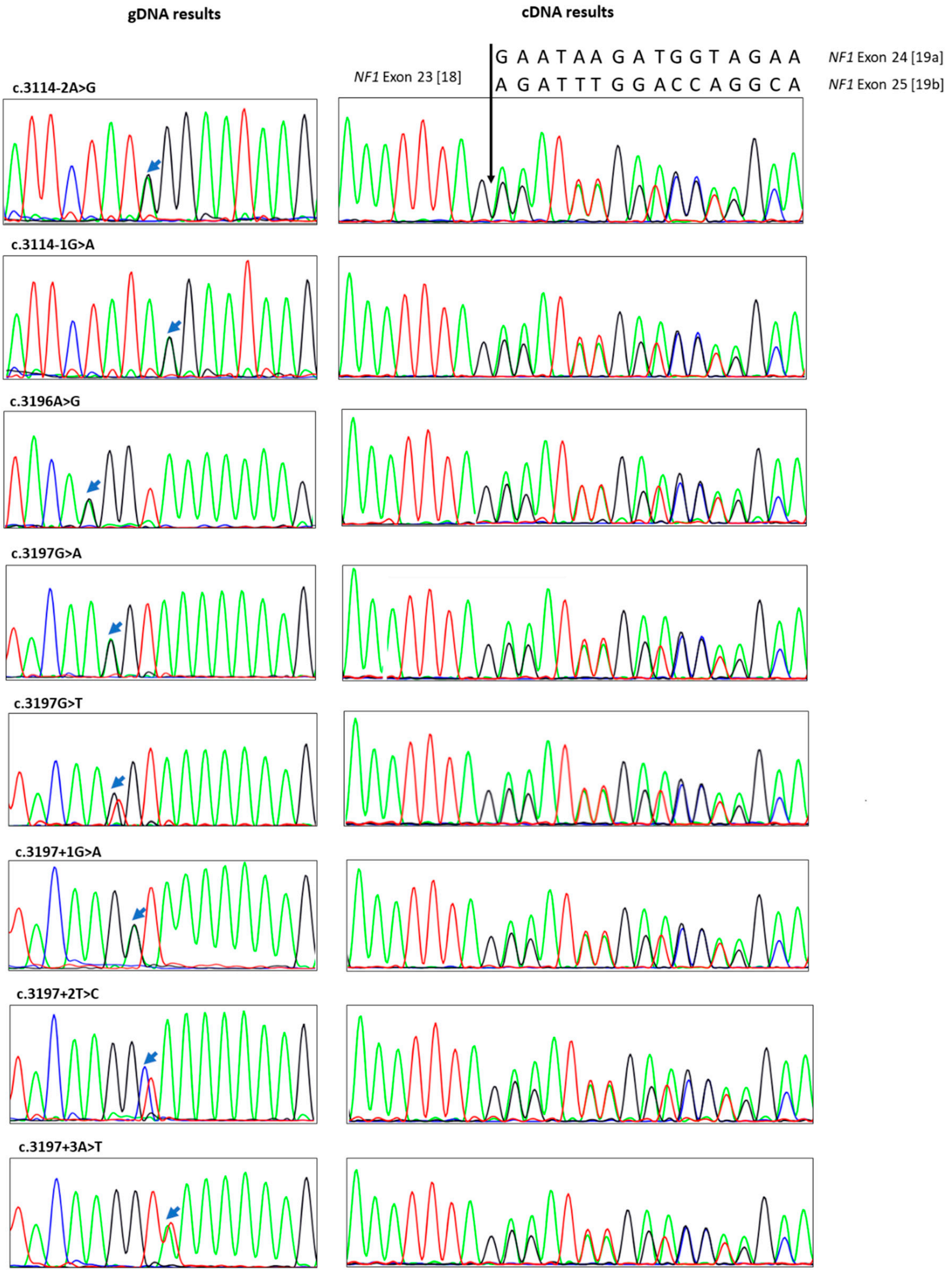

Figure S1. Sanger sequencing results of eight *NF1* variants that result in exon 24 [19a] skipping. The blue arrows indicate single nucleotide substitutions at the genomic DNA (g-DNA) level, and the black arrow indicates the boundary between *NF1* exon 23 [18] and exon 24 [19a]/exon 25 [19b] at the RNA/cDNA level.

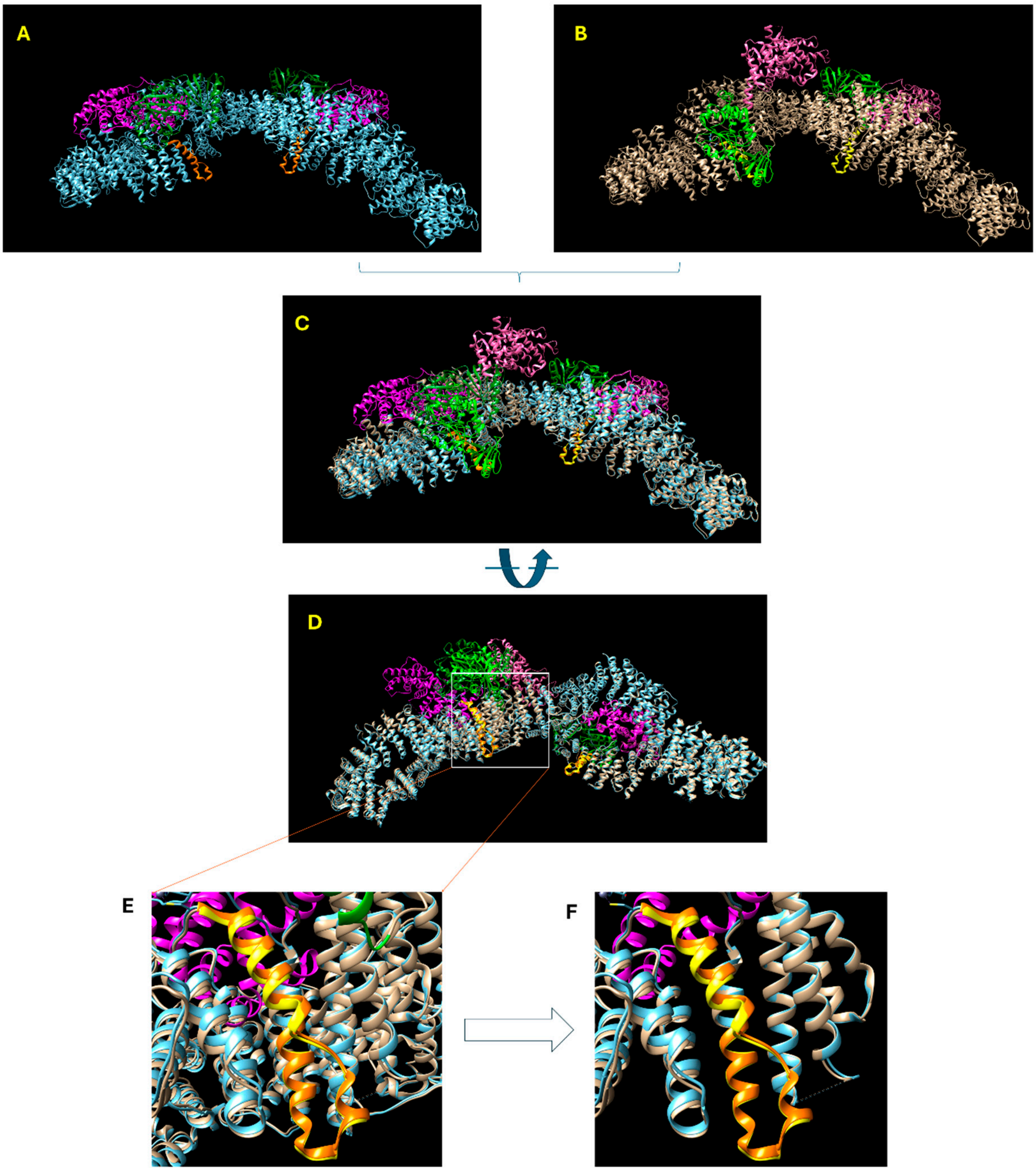

Figure S2. Location of the region of *NF1:*p.Asn1039_Arg1066 (deleted due to *NF1* exon 24 [19a] skipping), as well as flanking residues (p.Gln1033_Arg1038 and p.Asp1067_Met1073), in the 3D structures of human neurofibromin dimers, depicted by UCSF Chimera. **A.** The 3D structure of the human neurofibromin dimer in the closed state (PDB ID: 7PGR), highlighting the region p.Gln1033_Met1073 in orange, the GRD domain in purple, and the SEC14-PH domain in dark green. **B.** The 3D structure of the human neurofibromin dimer in the open state (PDB ID: 7PGT), highlighting the region p.Gln1033_Met1073 in yellow, the GRD domain in pink, and the SEC14-PH domain in light green. **C.** Overlay of the close and open states of 3D structures of neurofibromin dimers. The overlay demonstrates significant domain movements at the top of the dimer when it shifts from a closed state to an open state, whereas the bottom parts of the dimer have no noticeable structure change. **D.** Slight shift of the overlaid structures to expose the region p.Gln1033_Met1073. **E.** Zoomed-in result of the overlaid structures of the region p.Gln1033_Met1073. **F.** Zoomed-in result of the overlaid structures of the region p.Gln1033_Met1073 with some interference from other regions of neurofibromins hidden.

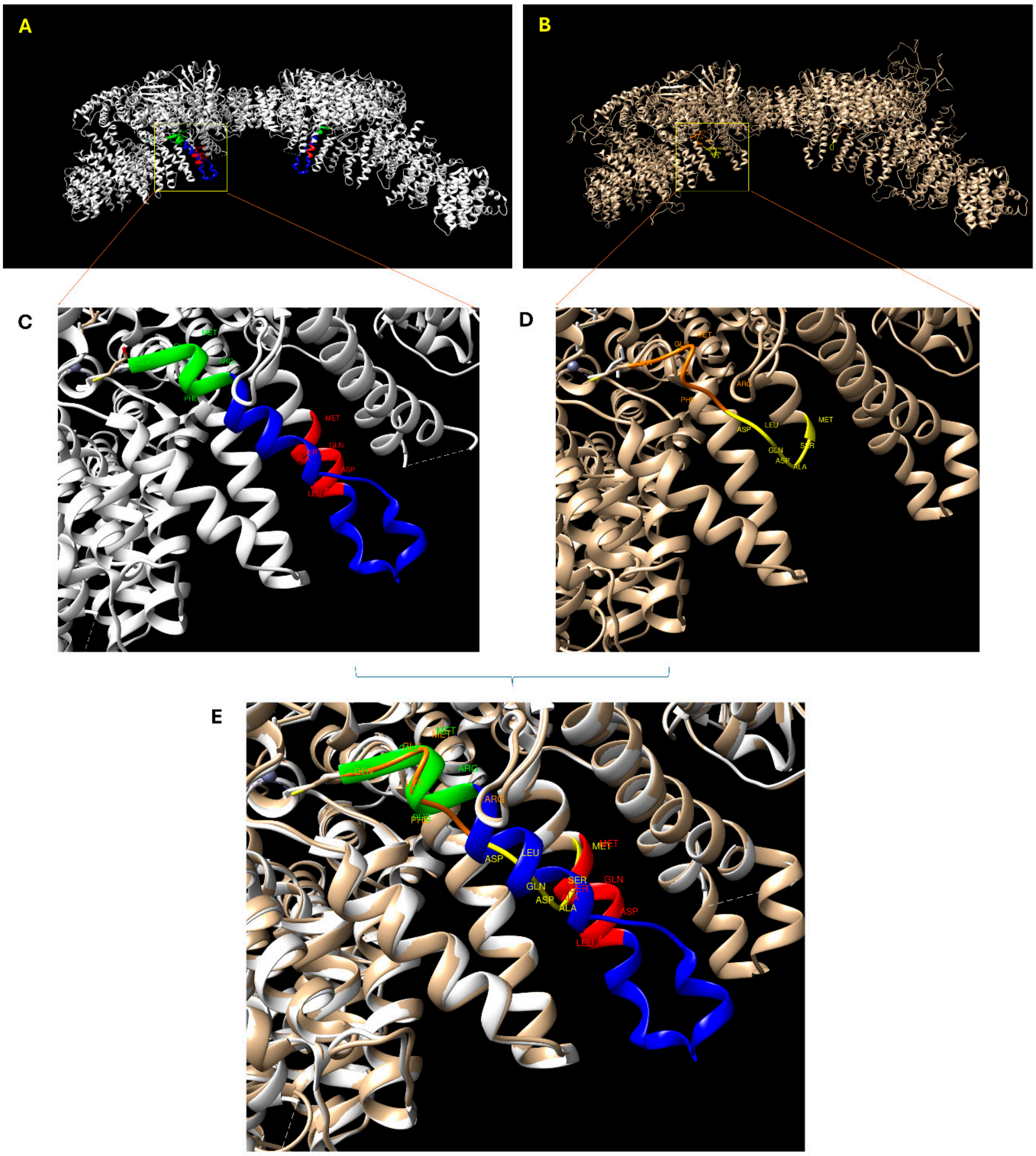

Figure S3. The potential effect of the deletion of the region of p.Asn1039_Arg1066 on the 3D structures of human neurofibromin dimers. **A.** The 3D structure of the human neurofibromin dimer in the closed state (PDB ID: 7PGR), highlighting the p.Asn1039_Arg1066 region in blue, the flanking region p.Gln1033_Met1038 in green, and the flanking region p.Asp1067_Met1073 in red. **B.** The homology modeled 3D structure of the human neurofibromin dimer lacking the p.Asn1039_Arg1066 region (by SWISS-MODEL using PDB ID: 7PGR as the template), highlighting the flanking region p.Gln1033_Met1038 in orange and the flanking region p.Asp1067_Met1073 in yellow. **C and D.** Zoomed-in results of the p.Gln1033_Met1073 region in the closed state of the human neurofibromin dimer and homology modeled human neurofibromin dimer (lacking the p.Asn1039_Arg1066 region). **E.** The overlaid structures for the closed state of the human neurofibromin dimer and homology modeled human neurofibromin dimer (lacking the p.Asn1039_Arg1066 region), focusing on the regions at and spatially near p.Asn1039_Arg1066.

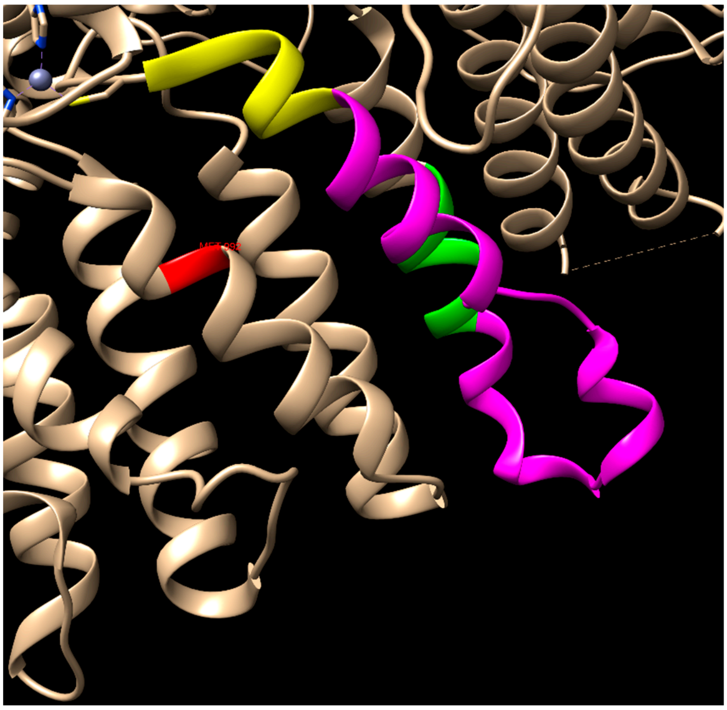

Figure S4. Location of Met992 (highlighted in red) and the region of *NF1*:p.Asn1039_Arg1066 (highlighted in purple), as well as flanking residues p.Gln1033_Arg1038 (highlighted in yellow) and p.Asp1067_Met1073 (highlighted in green), in the 3D structures of human neurofibromin dimers, depicted by UCSF Chimera.

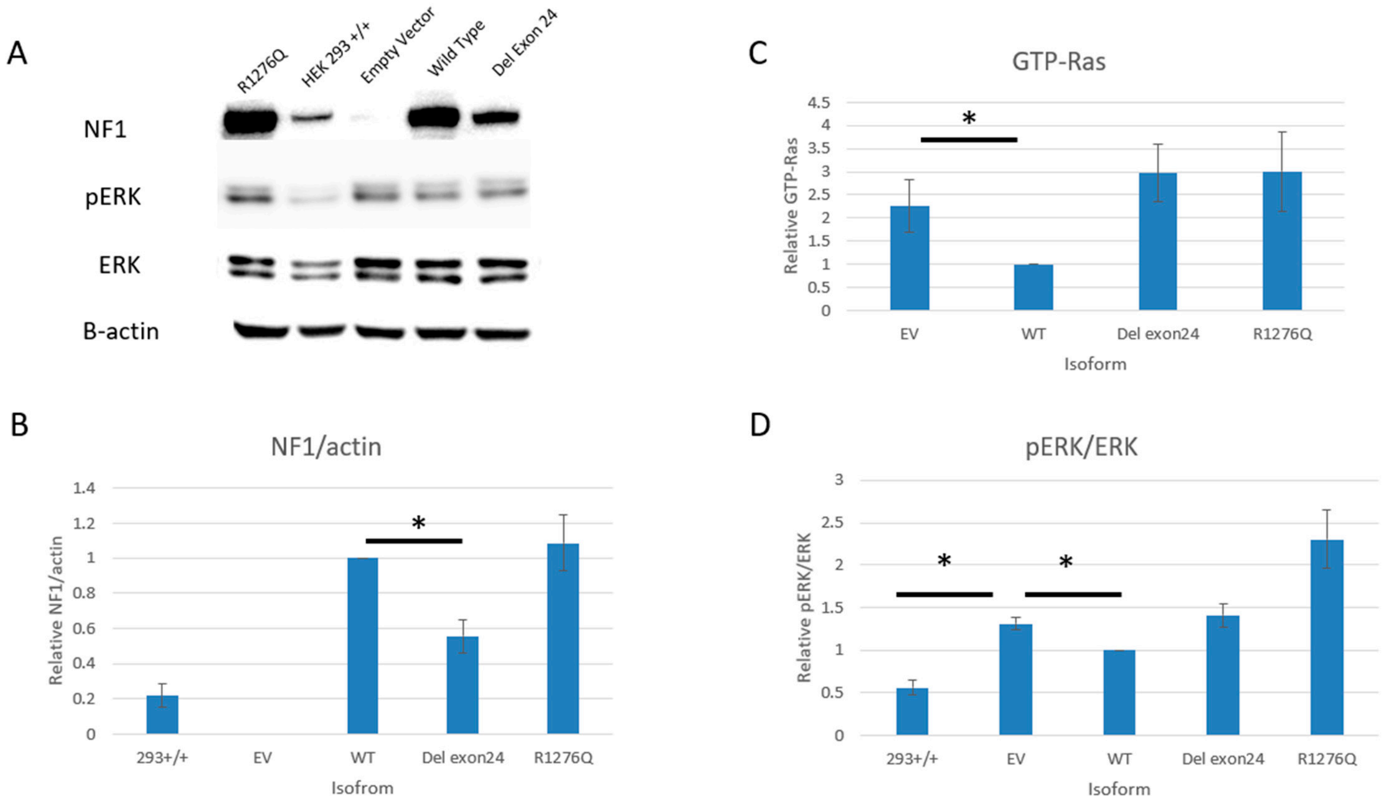

Figure S5. Functional analysis of mNf1 isoforms with selected variants: NF1 protein expression and RAS activity. (A) Representative western blot of NF1 protein (neurofibromin) and actin levels as well as pERK and ERK. (B) Quantitation of NF1/actin ratios normalized to WT ratio; N ≥ 3. Error bars represent SEM. Resultant neurofibromin abundance for each cDNA was compared with WT levels by t test. Asterisk indicates $p < 0.05$. (C) GTP-RAS levels normalized to WT and compared with EV control; N > 3. (D) Quantitation of pERK/ERK ratios normalized to WT and compared with EV control; N ≥ 3. Error bars represent SEM.

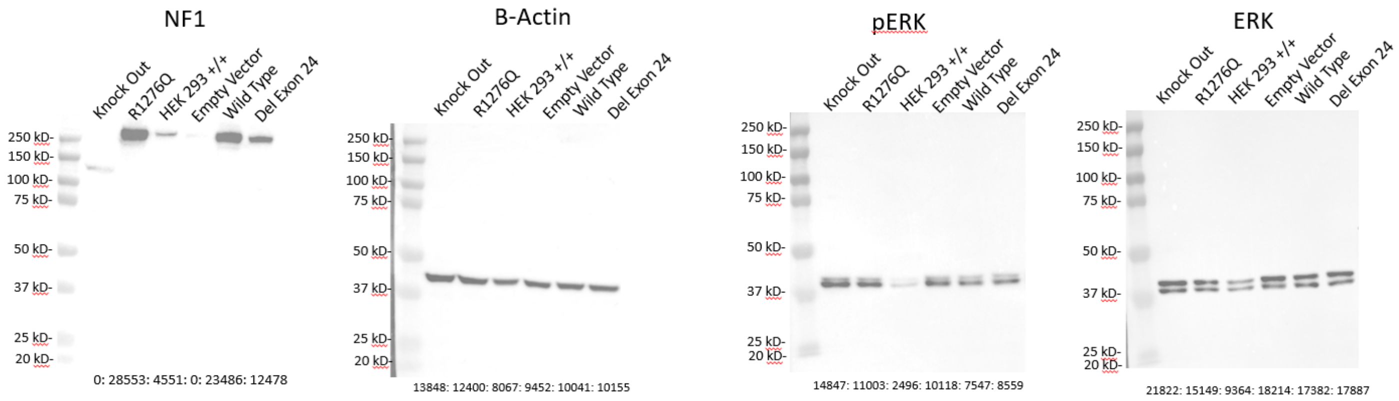

Figure S5 Full Blots with marker lables
